# Comparative transcriptome analysis reveals gene network regulating cadmium uptake and translocation in peanut roots under iron deficiency

**DOI:** 10.1186/s12870-019-1654-9

**Published:** 2019-01-21

**Authors:** Chu Chen, Qiqi Cao, Qun Jiang, Jin Li, Rugang Yu, Gangrong Shi

**Affiliations:** College of Life Sciences, Huaibei Normal University, Huaibei, Anhui 235000 People’s Republic of China

**Keywords:** Peanut, Iron deficiency, Cadmium, Transcriptome, Gene expression

## Abstract

**Background:**

Iron (Fe) is an essential element for plant growth and development, whereas cadmium (Cd) is non-essential and highly toxic. Previous studies showed that Fe deficiency enhanced Cd uptake and accumulation in peanuts. However, the molecular mechanism underlying the increased Cd accumulation in Fe-deficient peanut plants is poorly understood.

**Results:**

We employed a comparative transcriptome analysis approach to identify differentially expressed genes (DEGs) in peanut roots exposed to Fe-sufficient without Cd, Fe-deficient without Cd, Fe-sufficient with Cd and Fe-deficient with Cd. Compared with the control, Fe deficiency induced 465 up-regulated and 211 down-regulated DEGs, whereas the up- and down-regulated DEGs in Cd exposed plants were 329 and 189, respectively. Under Fe-deficient conditions, Cd exposure resulted in 907 up-regulated DEGs and 953 down-regulated DEGs. In the presence of Cd, Fe deficiency induced 1042 up-regulated and 847 down-regulated genes, respectively. Based on our array data, we found that metal transporter genes such as *CAX4*, *COPT1*, *IRT1*, *NRAMP5*, *OPT3*, *YSL3*, *VIT3* and *VIT4* might be involved in iron homeostasis. Moreover, combined with quantitative real-time PCR, *IRT1*, *NRAMP3*, *NRAMP5*, *OPT3*, *YSL3*, *ABCC3*, *ZIP1*, and *ZIP5* were verified to be responsible for Cd uptake and translocation in peanut plants under iron deficiency. Additionally, a larger amount of ABC transporter genes was induced or suppressed by iron deficiency under Cd exposure, indicating that this family may play important roles in Fe/Cd uptake and transport.

**Conclusions:**

The up-regulated expression of *NRAMP5* and *IRT1* genes induced by iron deficiency may enhance Cd uptake in peanut roots. The decrease of Cd translocation from roots to shoots may be resulted from the down-regulation of *ZIP1*, *ZIP5* and *YSL3* under iron deficiency.

**Electronic supplementary material:**

The online version of this article (10.1186/s12870-019-1654-9) contains supplementary material, which is available to authorized users.

## Background

Cadmium (Cd) is a non-essential and highly toxic heavy metal that is commonly released into the arable soil due to anthropogenic activities. The concentration of Cd in leaves greater than 5–10 μg g^− 1^ dry mass (DM) is toxic to non-tolerant crop plants [1]. For humans and animals, Cd may damage the mitochondrial and induce cell death by apoptosis and/or necrosis, leading to tissue inflammation and fibrosis [2]. Cd exposure has been shown to be associated with cancers of the prostate, lungs and testes in humans [3]. Due to its highly bioavailability, Cd is easily absorbed and accumulated in plants, and subsequently transferred to humans/animals via food chains. Thus, the presence of Cd in the arable soil can cause serious risks to human health. The minimization of Cd in edible parts of crops is an important demand especially in Cd-contaminated soil.

Iron (Fe) is an essential element that has multiple physiological functions including chlorophyll biosynthesis, photosynthesis, respiration, and redox reactions in plants. Despite its abundance in the earth’s crust, Fe often precipitates into insoluble Fe(III) oxides under aerobic conditions, especially in high-pH and calcareous soils [4]. Consequently, dissolved Fe in the soil solution is usually far lower than that required to sustain plant growth [5]. Iron deficiency causes chlorosis, growth retardation, and reduced crop productivity. It has become an important yield-limiting factor for crops growing in calcareous and alkaline soils.

Peanut (*Arachis hypogaea* L.) is one of the world’s fourth largest oilseed crop that is sensitive to Fe deficiency [6]. Peanut was also demonstrated to have a high capacity for accumulating Cd in both the seed and vegetative tissues depending on cultivars [7–9]. In previous studies, we have found that the uptake and accumulation of Cd in peanut plants is considerably enhanced by iron deficiency [10–13]. However, the mechanism underlying iron deficiency-induced increase of Cd accumulation in peanuts has not been well understood.

Generally, the accumulation of Cd in the shoot of plants is controlled by several biological processes, including (i) symplastic uptake by root epidermal cells, (ii) radial transport to the vascular cylinder, (iii) xylem loading, and (iv) root-to-shoot translocation [11]. Most of these processes are regulated by the transporters of several essential metals such as Fe, Mn, and Zn. During the past few years, several Fe transporters belonging to natural resistance associated macrophage proteins (Nramp), Zrt/Irt-like proteins (ZIP) and P1B-ATPases, have been identified to be responsible for the transport of Cd in plants [14]. Thomine et al. [15] found that iron deficiency could induce the expression of *AtNramp3* and *AtNramp4* in *Arabidopsis*. The increase of Cd uptake and accumulation under iron deficiency has been confirmed to be mediated by Fe^2+^ transporters such as OsIRT1 and OsIRT2 in rice [16]. Similarly, our results showed that the expression of *AhIRT1* and *AhNramp1* genes in peanut roots can be induced by iron deficiency, which is associated with Cd uptake and accumulation [13]. He et al. [17] demonstrated that Fe supply prevents Cd uptake in *Arabidopsis* by inhibiting *AtIRT1* expression and enhancing antagonism between Fe and Cd uptake.

Taken together, we hypothesize that iron deficiency-induced expression of Fe^2+^ transporter genes may be responsible for the increased uptake and accumulation of Cd in iron-deficient peanut plants. To test this hypothesis, a comparative transcriptome analysis was carried out on iron-sufficient and -deficient peanut plants under Cd exposure. The aims were: (i) to obtain the gene expression patterns in the roots under iron deficiency; (ii) to identify the key genes related to Fe/Cd uptake and translocation; and (iii) to elucidate the gene regulatory network that are responsible for Cd uptake and translocation in peanut plants under iron deficiency. The results presented here will be useful for better understanding the mechanism of Cd accumulation induced by Fe deficiency and facilitate gene function studies in peanut.

## Results

### The accumulation and translocation of cd and Fe in plants

**Fig. 1:**
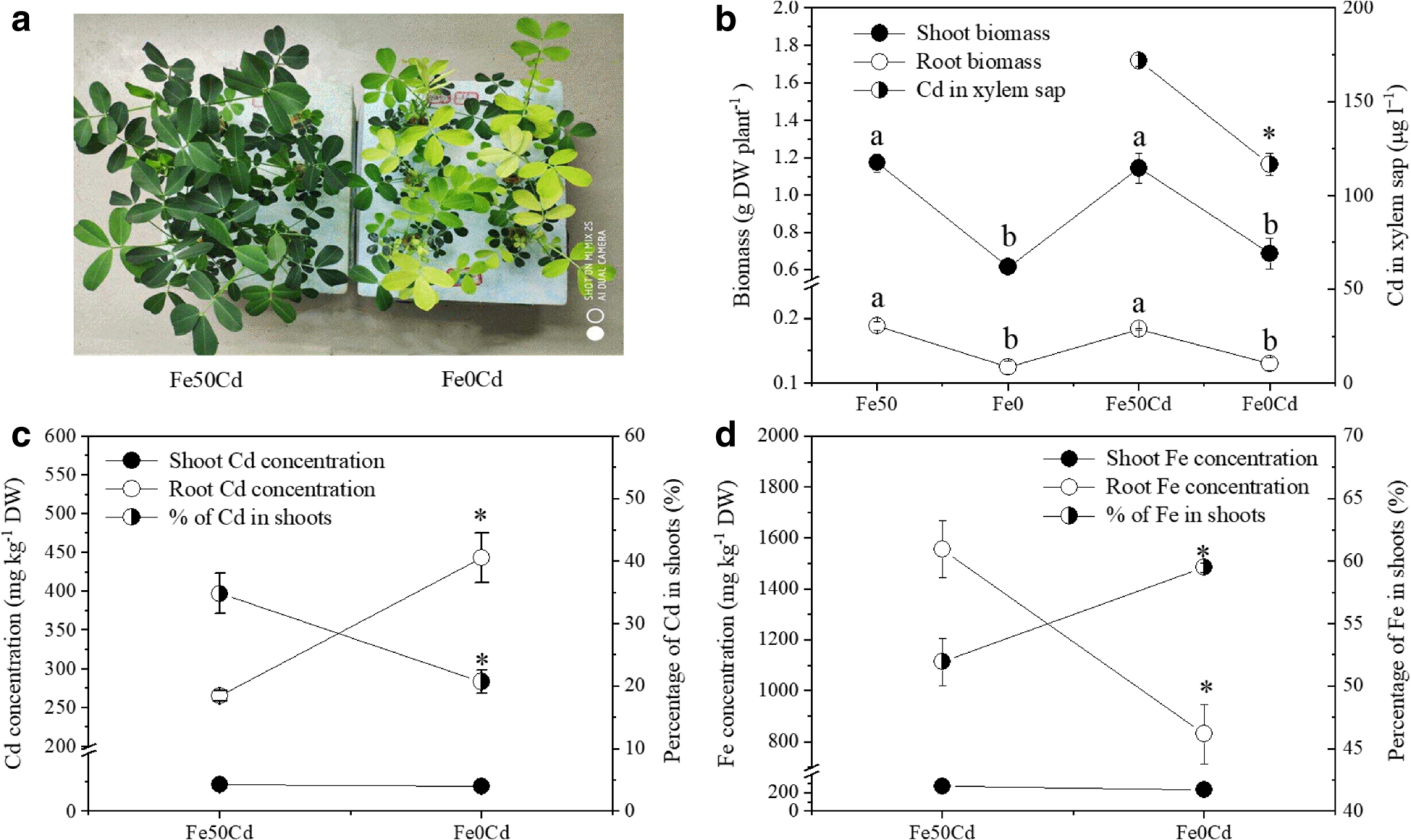
Growth (**a** and **b**) and the accumulation and translocation of Cd (**c**) and Fe (**d**) in Fe-sufficient (Fe_50_Cd) and Fe-deficient (Fe_0_Cd) peanut plants exposed to 2 μM Cd for seven days. Asterisk (*) above error bars indicate values (mean ± SE, *n* = 3) are significantly different between Fe-sufficient and Fe-deficient plants according to independent-samples t-test at 0.05 the level. Different letters above error bars indicate significant difference at 0.05 the level according to ANOVA followed by a Duncan multiple-range test

Figure 1 shows plant growth as well as the accumulation and translocation of Cd and Fe in peanut plants. Compared with normal Fe supply, Fe-deficiency inhibited plant growth and resulted in leaf chlorosis (Fig. 1 a and b), while Cd did not change plant growth in both Fe treatments (Fig. 1b). It was also observed that Fe-deficiency significantly increased root Cd concentrations but decreased Cd concentration in xylem sap and the percentage of Cd in shoots (Fig. 1 b and c), whereas Cd concentrations in the shoot were not affected (Fig. 1c). These results showed good agreement with previous findings [10–13], suggesting that Fe deficiency increased uptake but decreased root-to-shoot translocation of Cd in peanut plants. In comparison with Fe-deficient plants, Fe-sufficient plants exhibited lower root Fe concentration and higher proportion of Fe in shoots (Fig. 1d). The reverse trend in the percentage of Cd and Fe in shoots indicates that the two ions might compete with each other during the translocation from roots to shoots.

### RNA sequencing analysis of eight cDNA libraries

To assess the global transcriptome profile of peanut roots in response to Fe-deficiency and/or Cd exposure, RNA-Seq analysis was performed on peanut roots exposed to Fe-sufficient without Cd (Fe_50_), Fe-deficient without Cd (Fe_0_), Fe-sufficient with Cd (Fe_50_Cd) and Fe-deficient with Cd (Fe_0_Cd). Two biological replicates were performed per treatment, with a total of eight cDNA libraries constructed. A total of 58,774,869, 54,456,644, 55,033,082, and 58,221,919 raw reads were produced from two biological replicate libraries of Fe_50_, Fe_0_, Fe_50_Cd, and Fe_0_Cd respectively. After removing low quality reads and reads containing adapter sequences, a total of 57,331,325, 53,124,279, 54,059,155, and 56,589,717 clean reads remained for Fe_50_, Fe_0_, Fe_50_Cd, and Fe_0_Cd, respectively. The percentage of clean reads in all eight libraries was more than 97.14%, and the percentage of Q20 exceeded 97.44% (Table 1). Pearson’s correlation coefficients for all biological replicates were 0.949–0.959 (Additional file 1: Table S1), suggesting the data were highly reproducible.

**Table 1:** Overview of raw and clean reads in Fe-sufficient (Fe_50_) and Fe-deficient (Fe_0_) peanut plants exposed to 0 or 2 μM CdCl_2_ (Cd) for seven days

Sample	Total raw reads	Total clean reads	Total clean bases (gb)	Clean reads q20 (%)	Clean reads q30 (%)	Clean reads in raw reads (%)	GC content (%)
Fe50_1	28,406,266	27,712,721	8.31	0.02	97.56	93.09	97.56
Fe50_2	30,368,603	29,618,604	8.89	0.02	97.64	93.33	97.53
Fe0_1	28,516,330	27,732,406	8.32	0.02	97.63	93.3	97.25
Fe0_2	25,940,314	25,391,873	7.62	0.02	97.44	92.86	97.89
Fe0Cd_1	28,152,785	27,619,944	8.29	0.02	97.61	93.23	98.11
Fe0Cd_2	26,880,297	26,439,211	7.93	0.02	97.69	93.47	98.36
Fe50Cd_1	33,142,927	32,227,575	9.67	0.02	97.68	93.43	97.24
Fe50Cd_2	25,078,992	24,362,142	7.31	0.02	97.63	93.31	97.14

The high-quality clean reads were mapped to the *A. duranensis* genome (reference genome) using HISAT (Hierarchical indexing for spliced alignment of transcripts). Ultimately, more than 79% of the clean reads were successfully mapped for all cDNA libraries, and over 77% were observed to be unique mapped reads (Table 2), suggesting that the samples were comparable.

**Table 2:** Mapping results of clean reads against the peanut genomic sequence

sample	Total clean reads	Total mapped reads	Uniquely mapped reads	Multiple mapped reads	Spliced reads	Unspliced reads	Properly mapped reads
Fe50_1	55,425,442	43,980,460 (79.35%)	42,937,535 (77.47%)	1,042,925 (1.88%)	16,275,516 (29.36%)	26,662,019 (48.1%)	38,544,100 (69.54%)
Fe50_2	59,237,208	46,911,319 (79.19%)	45,798,117 (77.31%)	1,113,202 (1.88%)	17,286,768 (29.18%)	28,511,349 (48.13%)	41,375,852 (69.85%)
Fe0_1	55,464,812	45,419,922 (81.89%)	44,410,704 (80.07%)	1,009,218 (1.82%)	16,915,252 (30.5%)	27,495,452 (49.57%)	40,481,232 (72.99%)
Fe0_2	50,783,746	40,804,086 (80.35%)	39,847,134 (78.46%)	956,952 (1.88%)	14,885,388 (29.31%)	24,961,746 (49.15%)	36,103,056 (71.09%)
Fe0Cd_1	55,239,888	44,105,861 (79.84%)	42,995,894 (77.83%)	1,109,967 (2.01%)	16,183,762 (29.3%)	26,812,132 (48.54%)	38,747,872 (70.14%)
Fe0Cd_2	52,878,422	42,298,150 (79.99%)	41,248,604 (78.01%)	1,049,546 (1.98%)	15,493,356 (29.3%)	25,755,248 (48.71%)	37,208,590 (70.37%)
Fe50Cd_1	64,455,150	51,650,748 (80.13%)	50,399,248 (78.19%)	1,251,500 (1.94%)	18,847,959 (29.24%)	31,551,289 (48.95%)	45,817,274 (71.08%)
Fe50Cd_2	48,724,284	38,614,623 (79.25%)	37,716,105 (77.41%)	898,518 (1.84%)	13,970,730 (28.67%)	23,745,375 (48.73%)	34,228,200 (70.25%)

### Identification of differentially expressed genes (DEGs)

**Fig. 2:**
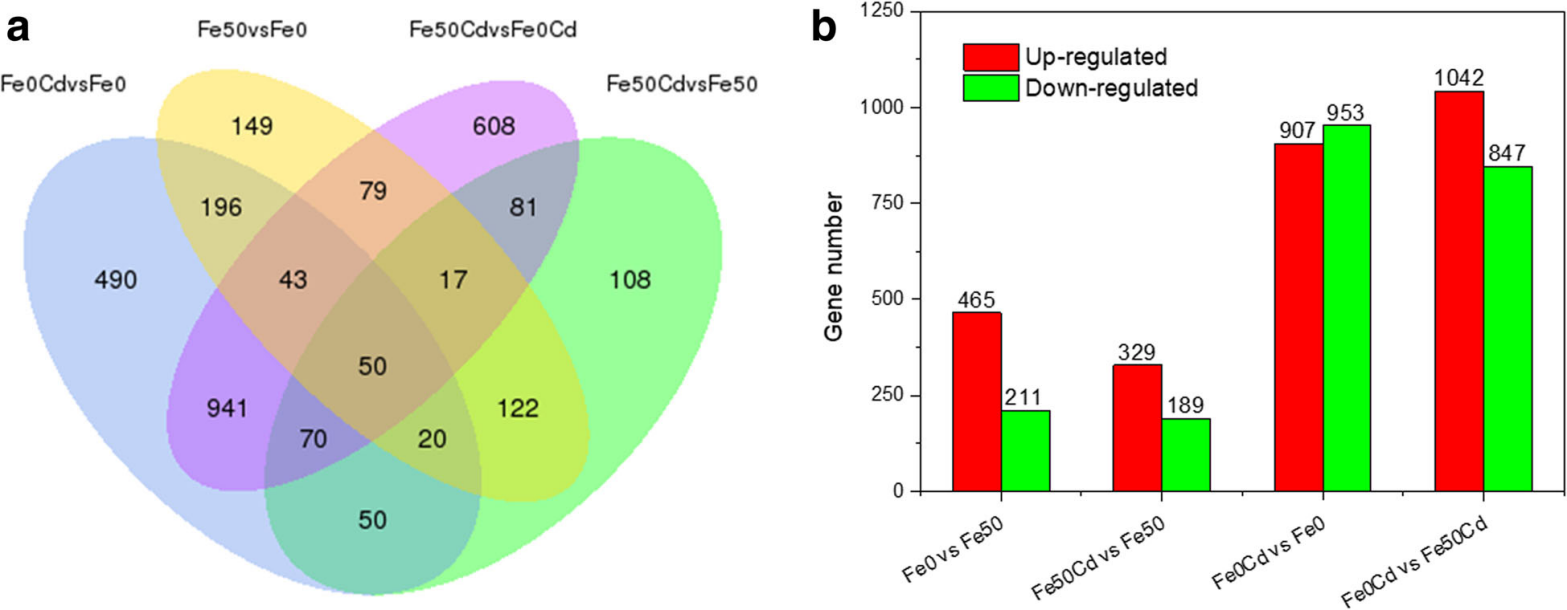
Gene expression profile of in Fe-sufficient (Fe_50_) and Fe-deficient (Fe_0_) peanut plants exposed to 0 or 2 μM CdCl_2_ (Cd) for seven days. (**a**) Venn diagram of DEGs. (**b**) The total number of up-regulated and down-regulated genes

A total of 63,191 genes including 34,553 known genes and 18,030 novel genes, 8017 lncRNA, 35 misc. RNA, 580 tRNA and 1976 pseudogenes were identified in eight cDNA libraries. Pairwise comparison analysis for each gene were performed between Fe-sufficient and Fe-deficient treatments (Fe_0_/Fe_50_ and Fe_0_Cd/Fe_50_Cd), or between Cd-absent and Cd-present treatments (Fe_50_Cd/Fe_50_ and Fe_0_Cd/Fe_0_). DEGs were identified by the threshold of *P*_adj_-value < 0.05. As a result, a total of 3024 genes were differentially regulated in the four comparisons, of which 676, 1889, 518, and 1860 DEGs were identified in Fe_0_ vs Fe_50_, Fe_0_Cd vs Fe_50_Cd, Fe_50_Cd vs Fe_50_ and Fe_0_Cd vs Fe_0_, respectively (Fig. 2a). Regardless of the absence or presence of Cd, 189 genes were differentially expressed between Fe-sufficient and Fe-deficient treatments, suggesting that these genes were specifically induced or supressed by Fe deficiency. It was also found that 190 genes were specifically regulated by Cd exposure under both Fe treatments (Fig. 2a).

Compared with the control (Fe_50_), Fe deficiency (Fe_0_) induced 465 up-regulated and 211 down-regulated DEGs, whereas the up- and down-regulated DEGs in Cd-exposed plants (Fe_50_Cd) were 329 and 189, respectively (Fig. 2b). Under Fe-deficient conditions, Cd exposure (Fe_0_Cd) resulted in 907 up-regulated DEGs and 953 down-regulated DEGs. In the presence of Cd, Fe deficiency (Fe_0_Cd) induced 1042 up-regulated and 847 down-regulated genes (Fig. 2b).

### Gene ontology (GO) analysis of DEGs

**Fig. 3:**
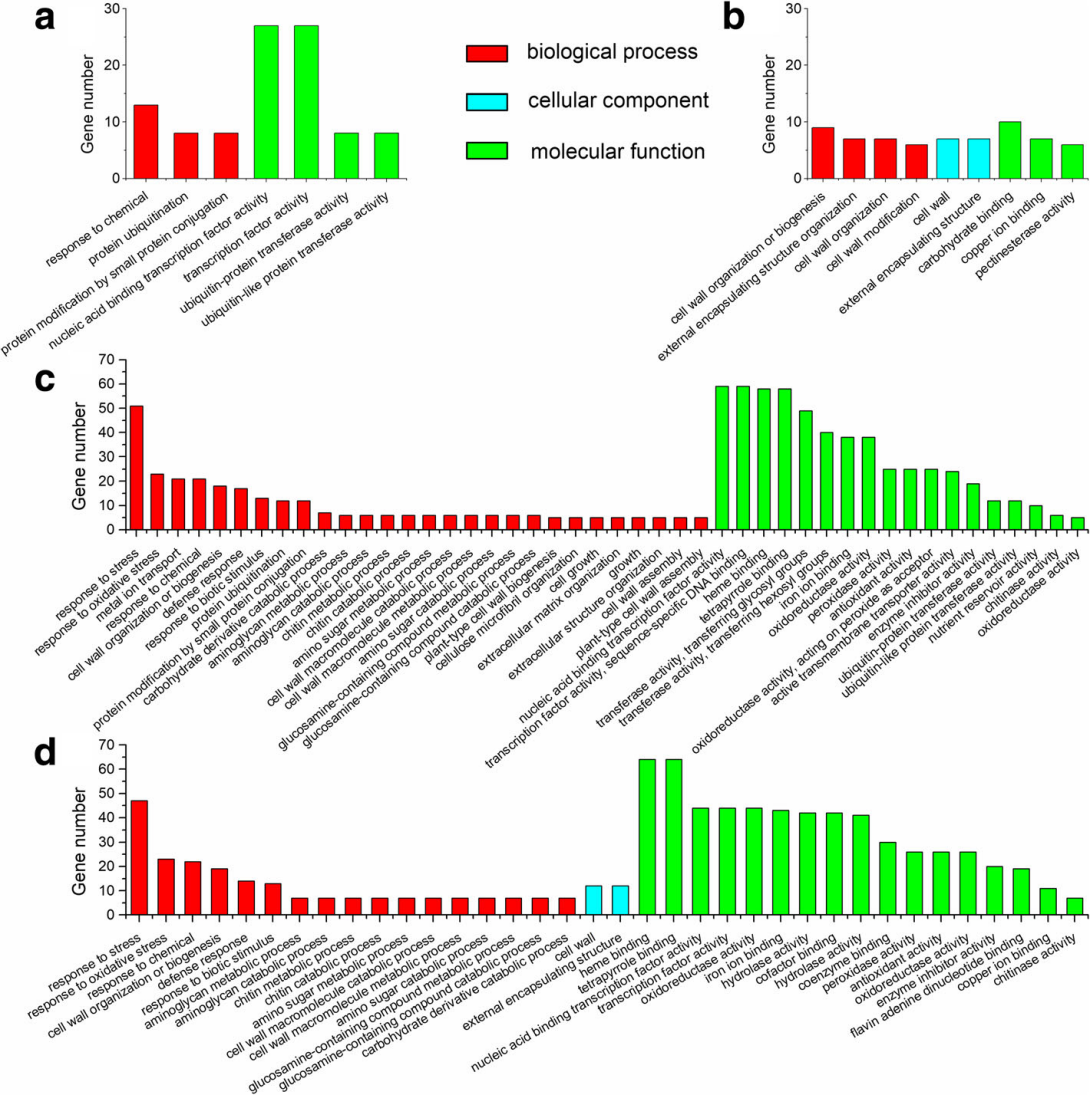
Gene ontology classification of DEGs identified. The enriched biological process, cellular component and molecular function GO terms of DEGs between Fe-sufficient and Fe-deficient peanut roots prior to (**a**) or after (**d**) Cd exposure, and between Cd-free and Cd-treated peanut roots under Fe-sufficient (**b**) and Fe-deficient (**c**) conditions

GO assignments were used to classify the functions of DEGs, and the results of significantly enriched GO terms (*P*_adj_-value < 0.05) were shown in Fig. 3. A total of 99 Fe deficiency-responsive DEGs (Fe_0_ vs Fe_50_) were assigned into 7 enriched GO terms consisting of 3 biological process (response to chemical, protein ubiquitination, protein modification by small protein conjugation) and 4 molecular function (nucleic acid binding transcription factor activity, transcription factor activity, ubiquitin-protein transferase activity, ubiquitin-like protein transferase activity) (Fig. 3a). Meanwhile, 66 Cd-responsive DEGs were assigned into 9 enriched GO terms, including 4 biological process (cell wall organization or biogenesis, external encapsulating structure organization, cell wall organization, cell wall modification), 2 cellular components (cell wall, external encapsulating structure) and 3 molecular function (carbohydrate binding, copper ion binding, pectinesterase activity) (Fig. 3b). A total of 857 DEGs between Fe_0_Cd and Fe_0_ were assigned into 46 enriched GO terms consisting of 28 biological process (response to stress, response to oxidative stress, metal ion transport, response to chemical, etc.) and 18 molecular function (nucleic acid binding transcription factor activity, transcription factor activity, heme binding, tetrapyrrole binding, etc.) subcategories (Fig. 3c), and 832 DEGs of Fe_0_Cd vs Fe_50_Cd were assigned into 36 enriched GO terms including 17 biological process (response to stress, response to oxidative stress, response to chemical, etc.), 2 cellular components (cell wall, external encapsulating structure) and 17 molecular function (heme binding, tetrapyrrole binding, etc.) subcategories (Fig. 3d).

### KEEG metabolic pathway analysis of DEGs

**Fig. 4:**
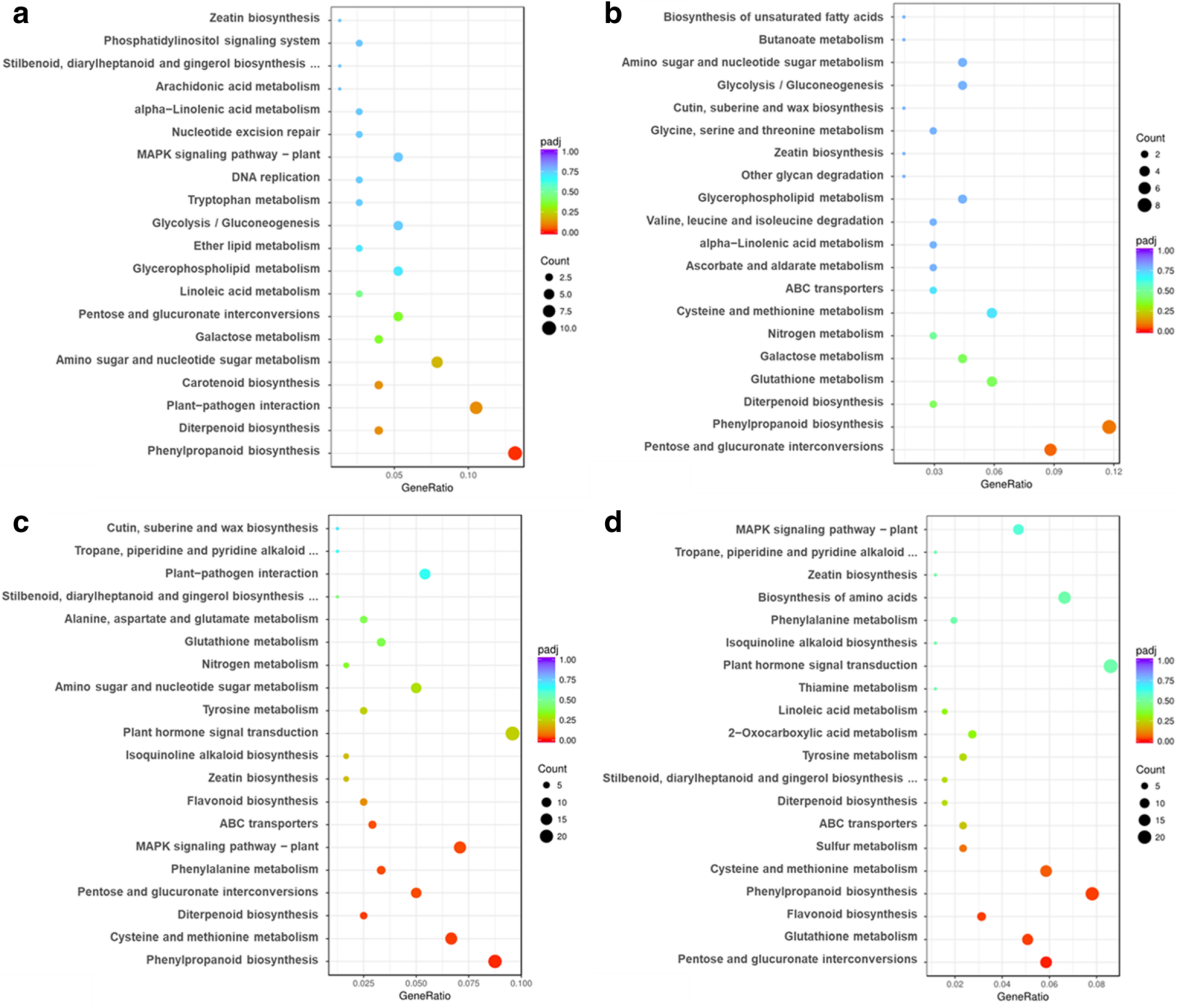
KEGG pathway enrichment analysis based on the differentially expressed genes between Fe-sufficient and Fe-deficient peanut roots prior to (**a**) or after (**c**) Cd exposure, and between Cd-free and Cd-treated peanut roots under Fe-sufficient (**b**) and Fe-deficient (**d**) conditions

To deep insight the molecular interactions among the genes, DEGs were further annotated with Kyoto Encyclopedia of Genes and Genomes (KEGG) database (http://www.genome.ad.jp/kegg/). A total of 76 (Fe_0_ vs Fe_50_), 256 (Fe_0_Cd vs Fe_50_Cd), 68 (Fe_50_Cd vs Fe_50_) and 240 (Fe_0_Cd vs Fe_0_) DEGs assigned to 48, 86, 47, and 88 pathways, respectively (Additional file 2: Table S2). The four comparisons differed from each other in metabolic pathways of DEGs (Fig. 4). The top five pathways of Fe deficiency-responsive DEGs were phenylpropanoid biosynthesis, plant-pathogen interaction, diterpenoid biosynthesis, carotenoid biosynthesis, and amino sugar and nucleotide sugar metabolism (Fig. 4a). Among these pathways, phenylpropanoid biosynthesis was identified as significantly enriched (*P*_adj_-value < 0.05). For Cd-responsive DEGs, pentose and glucuronate interconversions, phenylpropanoid biosynthesis, glutathione metabolism, galactose metabolism, and diterpenoid biosynthesis were the top five categories, and no enriched pathway was identified (Fig. 4b). A larger number of enriched pathways were found in DEGs of Fe_0_Cd vs Fe_50_Cd (pentose and glucuronate interconversions, glutathione metabolism, flavonoid biosynthesis, and phenylpropanoid biosynthesis) (Fig. 4c) and Fe_0_Cd vs Fe_0_ (phenylpropanoid biosynthesis, cysteine and methionine metabolism, diterpenoid biosynthesis, MAPK signaling pathway, pentose and glucuronate interconversions, phenylalanine metabolism, and ABC transporters) (Fig. 4d).

### DEGs involved in heavy metal transport

According to GO functional annotation, 54 DEGs were identified to highly similar with transporters that may be involved in the translocation of heavy metal in plants, including ATP-binding cassette transporters (ABC transporters), cation/H^+^ antiporters (CAXs), copper transport proteins (COPTs), Fe^2+^ transport proteins (IRTs), natural resistance-associated macrophage proteins (NRAMPs), metal tolerance proteins (MTPs), metal-nicotianamine transporter or yellow stripe-like transporters (YSLs), oligopeptide transporters (OTPs), vacuolar iron transporters (VITs), and zinc transporters (ZIPs) (Table 3). In the absence of Cd, *ABCB19*, putative *ABCC15*, *ABCG15*-like, *CAX4*-like, *COPT1*, *IRT1* (107484081 and 107494754), *NRAMP5* and *OPT3* were up-regulated by Fe deficiency, while three genes (*YSL3*, *VIT3* and *VIT4*) were down-regulated. In the presence of Cd, a total of 37 DEGs encoding metal transporters were detected between the Fe-sufficient and Fe-deficient peanut plants, including 18 up-regulated genes (*ABCA2*, *ABCA7*, *ABCB19*, *ABCB21*, *ABCB11*, *ABCC3*, *ABCC8*, *ABCC15*, *ABCF1*, *ABCG6*, *ABCG11*, *HMA5*, *CAX18*, *Nramp3*, *OPT3*, *IRT1*) and 19 down-regulated genes (*ABCB11*, *ABCB19*, *ABCG5*, *ABCG8*, *ABCG32*, *ABCG39*, *COPT6*, *OPT7*, *YSL3*, *YSL7*, *VIT4*, *VIT3*, *ZIP1*, *ZIP5*, *ZIP6*). Cd exposure induced seven metal transport genes in Fe-sufficient plants, including *ABCB11*, *ABCB19*, *IRT1* (110279939), *NRAMP5*, *HMA5*, *VIT4* (107468698) and *ZIP11*. In contrast, 32 transporter genes were induced or suppressed by Cd exposure under Fe deficient stress, including 19 up-regulated genes (*ZIP11*, *ABCC4*, *ABCC3*, *ABCC8*, *YSL3*, *ABCG11*, *CAX20*, *ABCG6*, *NRAMP3*, *HMA5*, *OPT3*, *ABCB11*, *CAX18*, *ABCG11*, *ABCC3*, *ABCB19*, *IRT1*) and 13 down-regulated genes (*ABCG15*, *CAX4*, *VIT3*, *COPT6*, *COPT1*, *COPT2*, *ABCB19*, *ABCG5*, *ABCB2*, *ABCB11*, *ZIP6*, *ABCG5*). Compared with the control, exposure of Fe-deficient plants to Cd up-regulated *ABCB11*, *ABCB19*, *ABCC3*, *ABCC8*, *ABCC15*, *ABCF1*, *ABCG6*, *ABCG11*, *CAX18*-like, *CAX20*, *IRT1*, *NRAMP3*, *NRAMP5*, *MTP11* and *OPT3*, but down-regulated *ABCG5*, *ABCG8*, *ABCG25*, *COPT1*, *VIT3*, *VIT4*, *ZIP1*, *ZIP5* and *ZIP6*. More importantly, it was found that three up-regulated genes (*OPT3*, *ABCC15*, *IRT1*) and three down-regulated genes (*VIT3*, *VIT4*, *YSL3*) were specifically regulated by Fe-deficiency, regardless of Cd treatments. Similarly, two genes (*ZIP11* and *HMA5*) were found to be specifically induced by Cd in both the Fe-sufficient and Fe-deficient peanut plants.

**Table 3:** DEGs possibly involved in metal transport in Fe-sufficient and Fe-deficient peanut plants exposed to 0 or 2 μM CdCl_2_ for seven days

Gene_ID	Gene description	Abbr.	Fe0vsFe50		Fe50CdvsFe50		Fe0CdvsFe50		Fe0CdvsFe0		Fe0CdvsFe50Cd	
			log2Fold Change	padj	log2Fold Change	padj	log2Fold Change	padj	log2Fold Change	padj	log2Fold Change	padj
107491333	ABC transporter A family member 2	ABCA2									0.753	0.013
107491332	ABC transporter A family member 7, transcript variant × 1	ABCA7									0.784	0.006
107479052	ABC transporter B family member 2-like, transcript variant X5	ABCB2							− 1.713	0.034		
107467550	ABC transporter B family member 11	ABCB11					2.600	0.000	2.761	0.000	2.126	0.000
107495097	ABC transporter B family member 11-like	ABCB11			1.352	0.009			−1.652	0.008	−2.400	0.000
107471179	ABC transporter B family member 19	ABCB19	1.270	0.013	1.005	0.010			−2.171	0.000	−1.889	0.000
107476483	ABC transporter B family member 19	ABCB19					2.380	0.000	3.120	0.000	1.561	0.020
107492327	ABC transporter B family member 21-like	ABCB21									2.000	0.030
107463694	ABC transporter C family member 3	ABCC3					1.313	0.000	1.045	0.004	1.339	0.000
107496250	ABC transporter C family member 3	ABCC3					3.254	0.000	3.073	0.000	2.248	0.000
107496270	ABC transporter C family member 3-like transcript variant ×2	ABCC3					−2.894	0.044				
107475935	ABC transporter C family member 4	ABCC4							0.890	0.020		
107464256	ABC transporter C family member 8, transcript variant ×2	ABCC8					1.918	0.000	1.132	0.010	1.548	0.000
107481980	putative ABC transporter C family member 15, transcript variant X2	ABCC15	2.018	0.000			2.888	0.000			2.391	0.000
107476479	ABC transporter F family member 1, transcript variant ×1	ABCF1					1.661	0.000			1.193	0.007
107470452	ABC transporter G family member 5-like	ABCG5					−1.958	0.000	−2.157	0.000	−1.762	0.000
107480222	ABC transporter G family member 5-like	ABCG5					−1.109	0.028	−1.497	0.002	−1.326	0.002
107467717	ABC transporter G family member 6-like	ABCG6					1.465	0.042	1.621	0.001	1.010	0.013
107466837	ABC transporter G family member 8-like	ABCG8					−1.145	0.041			− 1.331	0.001
107481537	ABC transporter G family member 11	ABCG11					1.012	0.028	1.305	0.001	1.107	0.002
107481662	ABC transporter G family member 11, transcript variant X2	ABCG11					4.356	0.000	3.055	0.000		
107492554	ABC transporter G family member 15-like	ABCG15	5.706	0.032					−5.572	0.019		
107467862	ABC transporter G family member 25	ABCG25					−1.042	0.018	−0.948	0.049		
107469510	ABC transporter G family member 32	ABCG32									−1.273	0.020
107493900	ABC transporter G family member 39-like	ABCG39									−2.463	0.017
110273497	Cation/H^+^ antiporter 4-like	CAX4	4.519	0.001					−4.765	0.001		
107479804	Cation/H^+^ antiporter 18-like, transcript variant X2	CAX18					1.903	0.000	2.838	0.000	2.334	0.000
107491686	Cation/H^+^ antiporter 20	CAX20					1.106	0.048	1.366	0.003		
107474428	Copper transport protein ATX1								0.981	0.006		
107477078	Copper transporter 1	COPT1	1.376	0.036			−1.926	0.002	−3.309	0.000		
107484791	Copper transporter 2-like, transcript variant ×1	COPT2							−2.964	0.000		
107484785	Copper transporter 6	COPT6							−3.354	0.026	−3.432	0.017
107484081	Fe^2+^ transport protein 1	IRT1	3.342	0.048			3.472	0.019				
107491017	Fe^2+^ transport protein 1	IRT1					9.778	0.000	7.200	0.000	9.773	0.000
107494754	Fe^2+^ transport protein 1-like	IRT1	6.608	0.023			11.806	0.000	5.210	0.000	8.432	0.000
110279939	Fe^2+^ transport protein 1-like	IRT1			6.658	0.020						
107486418	Metal tolerance protein 11, transcript variant x2	MTP11					0.890	0.046				
107480469	Metal transporter Nramp3, transcript variant ×1	Nramp3					2.912	0.000	1.898	0.000	3.565	0.000
107460699	Metal transporter Nramp5	Nramp5	1.945	0.000	1.633	0.001	1.325	0.001				
107460374	Metal-nicotianamine transporter	YSL3	−1.920	0.000					1.230	0.000	−0.648	0.042
107489952	Probable metal-nicotianamine transporter	YSL7									−0.901	0.012
107466086	Oligopeptide transporter 3	OPT3	1.857	0.000			4.594	0.000	2.725	0.000	4.720	0.000
107482451	Oligopeptide transporter 7, transcript variant x1	OPT7									−2.576	0.014
107471962	Probable copper-transporting ATPase	HMA5			1.311	0.000			2.409	0.000	1.311	0.000
107491634	Vacuolar iron transporter homolog 3	VIT3	−2.675	0.000			−6.523	0.001	−3.851	0.002	−6.562	0.001
107468698	Vacuolar iron transporter homolog 4	VIT4			2.925	0.032					−5.203	0.001
107481738	Vacuolar iron transporter homolog 4-like	VIT4					−1.108	0.005			−1.493	0.000
107481739	Vacuolar iron transporter homolog 4-like	VIT4	−4.145	0.000			−7.910	0.000			−8.370	0.000
107461527	Zinc transporter 1, transcript variant X1	ZIP1					−1.507	0.000			−1.671	0.000
107482454	Zinc transporter 5, transcript variant X2	ZIP5									−3.298	0.007
107494273	Zinc transporter 5-like	ZIP5					−6.223	0.021				
107458282	Zinc transporter 6, chloroplastic	ZIP6					−0.933	0.036				
107473088	Zinc transporter 6, chloroplastic	ZIP6					−1.994	0.000	−1.562	0.001	−1.418	0.006
107462023	Zinc transporter 11	ZIP11			0.925	0.009			0.831	0.034	−0.636	0.036

Data of DEGs showing a similar expression in pairwise comparisons were not shown in the table

### Verification of the DEG results

**Fig. 5:**
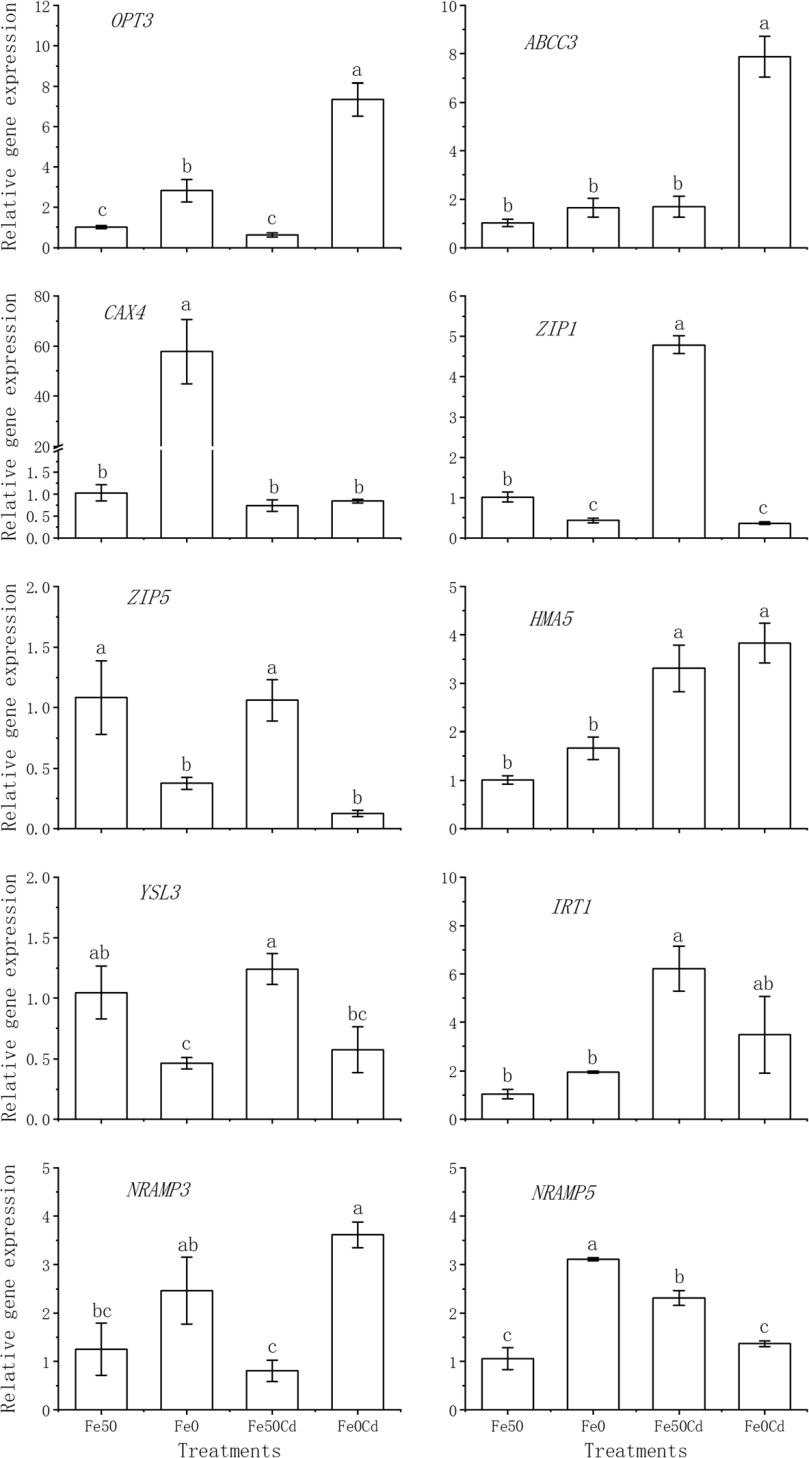
The qRT-PCR analysis of metal transport-related genes in peanut roots exposed to 0 or 2 μM Cd under Fe-sufficient (Fe50) and Fe-deficient (Fe0) conditions. The relative expression of each gene was calculated as the 2^−ΔΔCT^ value and normalized by geometric mean of three stably expressed reference genes. Data are presented as means with SE (*n* = 3). Different letters above error bars indicate that values are significantly different (*P* < 0.05) according to Duncan’s multiple range test after one-way analysis of variance

To further verify the transcriptome data, ten DEGs involved in metal transport, including *IRT1*, *NRAMP3*, *NRAMP5*, *OPT3*, *YSL3*, *CAX4*, *HMA5*, *ABCC3*, *ZIP1*, and *ZIP5*, were selected for RT-qPCR analysis. The RT-qPCR results, presented in Fig. 5, showed a good agreement with the RNA-Seq data (Table 3). Under Cd-free condition, Fe deficiency up-regulated the expression of *CAX4*-like, *NRAMP5* and *OPT3*, but down-regulated *YSL3*, *ZIP1* and *ZIP5* in peanut roots. For Fe-sufficient plants, the expression of *IRT1*, *NRAMP5*, *HMA5* and *ZIP1* was significantly induced by Cd exposure. Compared with the control, Cd exposure with Fe deficiency enhanced the expressions of *OPT3*, *ABCC3*, *HMA5*, and *NRAMP3*, but decreased those of *YSL3*, *ZIP1* and *ZIP5*. Pearson’s correlation analysis showed that the relative gene expression (RT-qPCR) is significantly correlated with the foldchange of read counts (RNA-Seq) (r = 0.705, *p* = 0.000, *n* = 40). The high confirmation rate demonstrates the reliability of our RNA-Seq data.

## Discussion

Although iron deficiency has been demonstrated to considerably enhance the uptake and accumulation of Cd in peanut plants [10–13], limited information is available about the physiological and molecular mechanisms underlying iron deficiency-induced increase of Cd accumulation in peanut. RNA-seq analysis has been used for revealing the molecular mechanisms of Cd uptake, translocation and accumulation in many plant species [18–20]. The complete genome sequencing of *A. duranensis* and *A. ipaensis*, the diploid ancestors of cultivated peanut [21], has shed light to genomic studies on cultivated peanut. However, sequence information of peanut in response to iron deficiency and Cd exposure is scarce. In this study, we obtained 57,331,325, 53,124,279, 54,059,155, and 56,589,717 clean reads from the peanut roots treated with Fe_50_, Fe_0_, Fe_50_Cd, and Fe_0_Cd respectively. More than 79% of clean reads for all cDNA libraries were functionally annotated in the *A. duranensis* genome [21], and more than 77% were unique mapped reads (Table 2). A total of 3024 genes were identified to be DEGs in pairwise comparisons: Fe_0_ vs Fe_50_ (676), Fe_0_Cd vs Fe_50_Cd (1889), Fe_50_Cd vs Fe_50_ (518), and Fe_0_Cd vs Fe_0_ (1860) (Fig. 2a). Of them, 54 DEGs were identified to highly similar with transporters that may be involved in the uptake and translocation of Fe/Cd in plants. These results provide clues to mechanisms underpinning Cd uptake and accumulation in Fe-deficient peanut plants.

Genes involved in Fe uptake and translocation were greatly induced by Fe deficiency in peanut roots. As an Fe-efficient plant that develops strategy I mechanism in response to Fe deficiency, peanut takes up Fe^2+^ by increasing Fe^2+^ transporter coupled with an increase of ferric reductase activity and rhizosphere acidification by releasing protons from the roots under Fe-deficient conditions [6]. We found that both the Fe^2+^ transport gene IRT1 and ferric reductase FRO1 were up-regulated by Fe deficiency in peanut roots (Table 3, Fig. 5). The result indicates that IRT1/FRO1 system constitutes the major pathway for Fe entry into root epidermal cells, and induction of these genes might improve Fe nutrition under Fe-deficient stress. A transporter gene of NRAMP family, *NRAMP5* (*NRAMP1*) [13], was also highly induced in roots under Fe-deficient conditions (Table 3, Fig. 5). The NRAMP5 (NRAMP1) have been shown to function in Fe transport in several plants such as *Arabidopsis* [22], *Noccaea caerulescens* [23], *Malus baccata* [24], peanut [25] and rice [26]. The induction of *NRAMP5* (*NRAMP1*) under Fe-deficiency indicates that this gene is involved in the uptake of Fe by peanut roots. Interestingly, both the IRT1 and NRAMP5 are shown to be involved in Cd transport into root cells across membrane [13, 16, 27]. The current results confirmed that iron deficiency-induced expression of IRT1 and NRAMP5 may be responsible for the increase of Cd uptake and accumulation in Fe-deficient peanut plants [13].

We also found that large number of ABC transporter genes were significantly induced or suppressed by Fe deficiency and/or Cd exposure (Table 3). Although the detailed functions of ABC transporter genes is poorly understood, some members have been verified to play roles in the uptake and translocation of Fe/Cd in plants [28]. ABCB19, an auxin transporter that mediates long-distance polar auxin transport in stems and roots [29, 30], was induced by Fe deficiency and/or Cd exposure (Table 3). Fe deficiency can increase the levels of auxin in the roots, which may promote the formation of root hair [31]. ABCC3 (MRP3), like most ABCC transporters such as such as ABCC2 and ABCC3, is vacuolar membrane-localized protein involved in the vacuolar transport of PC-Cd complexes [32]. The induction of ABCC3 in peanut roots by Fe deficiency with Cd exposure (Table 3, Fig. 5) may contribute to vacuolar Cd sequestration, enhancing Cd detoxification and reducing root-to-shoot translocation of Cd. *ABCC8* homologous to *MRP6* in Arabidopsis, which was shown to be part of a cluster (*AtMRP6*, *AtMRP3* and *AtMRP7*, as well as *SAT3*) involved in metal tolerance [33].

Besides ABC transporters, another peptide transporter OPT3 was significantly induced under Fe-deficient conditions (Table 3, Fig. 5). In Arabidopsis, OPT3 was shown to load iron into the phloem, facilitate iron recirculation from the xylem to the phloem, and regulate both shoot-to-root iron signaling and iron redistribution from mature to developing tissues [34, 35]. Reduced expression of OPT3 induces an over accumulation of Fe in roots and leaves, partially due to an elevated expression of the IRT1 [36]. Therefore, the strong induction of OPT3 in the root of Fe-deficient peanut seedlings suggests that the gene play a role in the redistribution of Fe between vegetative tissues. Moreover, OPT3 was also found to have an impact on the uptake and translocation of Cd in *Arabidopsis* [34, 35]. Mendoza-Cózatl et al. [35] observed that an OPT3 mutant of *Arabidopsis*, *opt3–2*, over-accumulates Cd in seeds and roots, but under-accumulates Cd in leaves. However, Zhai et al. [34] demonstrated that OPT3 is not involved in the phloem-based remobilization of Cd from source to sink tissues, despite it can mediate Cd transport in vitro. They speculated that increased Cd in seeds of the mutant is due to the impact of reduced expression of OPT3 on other transporters such as YSL1 or YSL3 [34]. YSL3 is a plasma-localized transporter delivering a broad range of nicotianamine-metal complexes. *SnYSL3* gene is shown to be involved in the translocation and detoxification of Cd in *Solanum nigrum*, and its overexpression in *Arabidopsis* increased the translocation of Cd and Fe from roots to shoots [37]. In the present study, YSL3 was down-regulated in peanut roots under Fe-deficient stress (Table 3, Fig. 5), which may be partially responsible for the reduced root-to-shoot translocation of Cd in Fe-deficient plants.

Several other transporter genes such as CAX4-like, COPT1, VIT3 and VIT4, were highly induced or suppressed by Fe deficiency (Table 3, Fig. 5). CAX4 is a cation/H^+^ antiporter that plays a key role in mediating cations, such as Ca^2+^ and Cd^2+^, influx into the vacuole [38]. Thus, the induction of CAX4-like by Fe deficiency in peanut roots may enhance vacuole sequestration of Cd and consequently, reducing root-to-shoot Cd translocation. COPT1 participates in copper acquisition and accumulation and regulates root elongation and pollen development [39]. In the current study, we found that COPT1 was up-regulated by Fe deficiency, but down-regulated in Fe-deficient seedlings exposed to Cd. We inferred that COPT1 indirectly effect Fe and/or Cd acquisition by regulating root elongation. Regardless of Cd exposure, the vacuolar iron transporters, VIT3 and VIT4, were suppressed under Fe deficiency condition. These alterations could reduce Fe sequestration in the vacuolar of root cells, and therefore enhance Fe transport from roots to shoots. NRAMP3 localizes at the vacuolar membrane, and is able to release Fe and Cd from the vacuolar under limited Fe conditions [40, 41]. The induction of NRAMP3 may improve the remobilization of Fe from vacuoles of root cells in Fe-deficient peanut plants particularly under Cd stress.

Apart from abovementioned genes, three genes belonging to ZIP family, ZIP1, ZIP5, and MTP11, differentially expressed between the control and Cd exposure with Fe deficiency (Table 3, Fig. 5). ZIP1 shows a high degree of homology to the AtZIP2 from Arabidopsis [42] and OsZIP1 from rice [43], while ZIP5 is homologous to AtZIP1 [42]. Both the AtZIP2 and OsZIP1 show a broad substrate specificity for divalent cations including Cd [42, 43]. In Arabidopsis, AtZIP2 is a plasma membrane localized transporter that may contribute to Mn/Zn movement in the stele to the xylem parenchyma, for subsequent xylem loading and transport to the shoot [44]. Similarly, *OsZIP1* transcripts were localized to the epidermis and stele of roots of zinc-deprived plants, suggesting the involvement of this transporter in zinc absorption or transfer from the vascular tissue [43]. AtZIP1 is a vacuolar transporter that may play a role in remobilizing Mn from the vacuole to the cytoplasm in root stellar cells, and may contribute to radial movement to the xylem parenchyma [44]. Thus, the reduced expression of ZIP1 and ZIP5 in the roots of Fe-deficient peanut plants under Cd stress may inhibit root-to-shoot Cd translocation. In the case of MTP11, it was shown to function in Mn transport and tolerance by sequestering Mn into the pre-vacuolar compartments in Arabidopsis [45]. Whether MTP11 is associated with Fe and/or Cd transport need to be further investigated.

## Conclusions

In conclusion, the current comparative study revealed that *CAX4*, *COPT1*, *IRT1*, *NRAMP3*, *NRAMP5*, *OPT3*, *YSL3*, *VIT3* and *VIT4* might be involved in iron homeostasis in Fe-deficient peanut plants. More importantly, some genes such as *IRT1*, *NRAMP3*, *NRAMP5*, *OPT3*, *YSL3*, *ABCC3*, *ZIP1*, and *ZIP5*, were identified to be responsible for the uptake, distribution, and translocation of Cd in peanut plants under iron deficiency. Based on our array data, we proposed a model to explain why iron deficiency induced an increase of Cd uptake but a decrease of Cd translocation from roots to shoots in peanut plants. The up-regulated expression of *NRAMP5* and *IRT1* genes induced by iron deficiency may enhance the uptake of cadmium by peanut roots. Iron deficiency-induced down-regulation of *ZIP1*, *ZIP5* and *YSL3* might result in a decrease of Cd xylem (or phloem) loading in root stele, and consequently, reducing root-to-shoot Cd translocation in peanut plants. Additionally, although detailed information is still unclear, larger amounts of ABC transporter genes were induced or suppressed by iron deficiency under Cd exposure, indicating that further study of this family would be helpful to understand the mechanism of Fe/Cd uptake and transport.

## Methods

### Plant growth and treatment

Peanut plants (*Arachis hypogaea* cv. Fenghua 1) were cultured as the conditions previously reported by Chen et al. [13] in a growth chamber of the Huaibei Normal University. Seeds (obtained commercially from the Peanut Institute of Shandong Province, Qingdao) were sterilized with 1% sodium hypochlorite for 10 min, and then they were rinsed with tap water for 24 h and germinated in well-washed sand. After 5 days of emergence, the uniform sized seedlings were selected and transferred to polyethylene pot (six plants per pot) containing 3.5 L of nutrient solution (pH 5.8) [11]. Seven-day-old seedlings were pretreated with (Fe_50_) or without (Fe_0_) 50 μM FeEDTA for 7 d in basal nutrient solution. Thereafter, 0 or 2 μM CdCl_2_ were added in nutrient solution for each Fe treatment, and continuously cultured for one week. The Cd level (2 μM) was arranged according to the Cd concentration in the soil solution of a Cd-contaminated farmland in China (soil moisture at 60% of field capacity) [7]. The experiment was arranged as a completely random design with nine replications (pots). During the growing period, the nutrient solution was renewed twice a week, and pots were randomly arranged and moved daily to minimize position effects.

Root samples for RNA-seq (two biological replicates) and qRT-PCR (three biological replicates) analysis were collected separately from Fe-sufficient and Fe-deficient plants after Cd treatment. Each biological replicate contains a pool of six different plants growing in each pot. All samples were immediately frozen in liquid nitrogen and stored at − 80 °C.

### Determination of cd and Fe in plants

Three pots of plants for each Fe treatment under Cd exposure were used for collecting xylem sap excluded from the cut surface as the method described by Uraguchi et al. [46]. After weighing, the collected sap was used for determining the Cd concentration by graphite furnace atomic absorption spectrometry (GF-AAS). Roots and shoots were separated and rinsed three times with deionized water. Thereafter, plant samples were oven-dried at 70 °C for constant weight. The dried tissues were weighed and ground into powder. The concentration of Cd and Fe in plant samples was measured by flame AAS after digested with HNO_3_–HClO_4_ (3:1, *v*/v).

The translocation of Cd and Fe from roots to shoots was indicated as the percentage of metal in shoots, which was calculated as follows [7]:

$$ \mathrm{Percentage}\ \mathrm{of}\ \mathrm{metal}\ \mathrm{in}\ \mathrm{shoot}\mathrm{s}\ \left(\%\right)=\kern0.5em 100\kern0.75em \times \kern0.5em \ {\left[\mathrm{M}\right]}_{\mathrm{shoot}}\times \mathrm{shoot}\ \mathrm{biomass}/\left({\left[\mathrm{M}\right]}_{\mathrm{root}}\times \mathrm{root}\ \mathrm{biomass}\kern0.5em +\kern0.5em {\left[\mathrm{M}\right]}_{\mathrm{shoot}}\times \mathrm{shoot}\ \mathrm{biomass}\right).\mathrm{where}\ \left[\mathrm{M}\right]\ \mathrm{is}\ \mathrm{the}\ \mathrm{concentration}\ \mathrm{of}\ \mathrm{Cd}\ \mathrm{or}\ \mathrm{Fe}\ \mathrm{in}\ \mathrm{plant}\ \mathrm{tissues} $$

### cDNA library construction and RNA sequencing

Total RNA was extracted by using Trizol® Reagent (Invitrogen) and purified using the RNeasy Plant Mini kit (Qiagen) according to the manufacturer’s instructions. The purity and integrity of RNA were analyzed using NanoPhotometer® spectrophotometer (IMPLEN, CA, USA) and Agilent 2100 Bioanalyzer (Agilent, USA), respectively. Eight cDNA libraries named Fe_50__1, Fe_50__2, Fe_0__1, Fe_0__2, Fe_50_Cd _1, Fe_50_Cd _2, Fe_0_Cd_1 and Fe_0_Cd_2 were constructed as the method previously described by Hu et al. [47]. The clustering of the index-coded samples was performed on a cBot Cluster Generation System using TruSeq PE Cluster Kit v3-cBot-HS (Illumia) according to the manufacturer’s instructions. After cluster generation, the library preparations were sequenced on an Illumina Hiseq™ 2500 platform and 125 bp/150 bp paired-end reads were generated. All library construction and sequencing were done at the Novogene Bioinformatics Institute (Beijing, China).

### Bioinformatics analysis

#### Data filtering and mapping

To obtain high-quality clean reads, the raw reads from RNA-seq were filtered by removing the adaptor sequences, ambiguous ‘N’ nucleotides and low-quality sequences. Clean reads were aligned to the peanut reference genome (NCBI) using HISAT2 (v2.2.4, http://www.ccb.jhu.edu/software/hisat) with default parameters. The mapped reads of each sample were assembled by StringTie (v1.3.3b) [48] in a reference-based approach.

#### Identification of DEGs

The numbers of reads mapped to the reference were counted using featureCounts v1.5.0-p3 [49]. The gene expression levels were represented by the expected number of Fragments Per Kilobase of transcript sequence per Millions base pairs sequenced (FPKM), which was calculated on the basis of the length of the gene and reads count mapped to this gene. Differential expression analysis of two groups was performed using the DESeq2 R package (1.16.1) according to the method described by Love et al. [50]. The resulting *P*-values were adjusted using the Benjamini and Hochberg’s approach for controlling the false discovery rate. Genes with an adjusted *P*_adj_-value < 0.05 found by DESeq2 were assigned as differentially expressed.

#### Gene ontology (GO) and pathway analysis of DEGs

Gene Ontology (GO) enrichment analysis of differentially expressed genes was implemented by the clusterProfiler R package, in which gene length bias was corrected.

GO terms with corrected *P* value less than 0.05 were considered significantly enriched by differential expressed genes. KEGG pathways were retrieved from KEGG web server (http://www.genome.jp/kegg/). The clusterProfiler R package was used to test the statistical enrichment of differential expression genes in KEGG pathways.

#### qRT-PCR validation

Ten DEGs were randomly selected for qRT-PCR validation. Primer sequences of these genes as well as reference genes are listed in Additional file 3: Table S3. Total RNA (0.5 μg) from each root sample was reverse transcribed into cDNA using Prime Script® RT reagent Kit (Takara, Dalian, China) and random primers following manufacturer’s instructions. Quantitative PCR reactions were performed in 20 μl reaction volumes using a SYBR Premix EX Taq Kit (Takara) according to the manufacturer’s instructions. Reactions were carried out on an ABI7300 (Applied Biosystems, CA, USA). Each biological replicate was technically replicated three times. The relative expression levels of the selected genes were calculated using the 2^-ΔΔ*C*T^ method and normalized by geometric mean of three stably expressed housekeeping genes (*AhADH3*, *Ah60S* and *Ahactin*) [51, 52].

## Additional files

Additional file 1: **Table S1.** Pearson correlation between samples. (DOCX 13 kb)

Additional file 2: **Table S2.** Overview of KEGG pathways for DEGs in four comparisons. (XLSX 21 kb)

Additional file 3: **Table S3.** The primers used in RT-qPCR analysis. (DOCX 15 kb)

## Abbreviations

ABC: ATP-binding cassette transporter protein
CAX: Cation/H+ exchanger
Cd: Cadmium
COPT: Copper transporter
DEG: Differentially expressed gene
GO: Gene ontology
HMA: Cadmium/zinc/copper-transporting ATPase (heavy metal-associated domain)
KEGG: Kyoto encyclopedia of genes and genomes
MTP: Metal tolerance protein
NA: Nicotianamine
NRAMP: Natural resistance-associated macrophage protein
OPT: Oligopeptide transporter
PC: Phytochelatin
qRT-PCR: Quantitative Real-time PCR
YSL: Yellow stripe-like transporter
ZIP: Zinc-regulated transporter/Iron-regulated transporter-like Protein
