# Supplementary material for: Comparative transcriptome analysis reveals gene network regulating cadmium uptake and translocation in peanut roots under iron deficiency
Source: BMC Plant Biol. 2019 Jan 21;19:35. doi: 10.1186/s12870-019-1654-9 (PMC6341601; doi:10.1186/s12870-019-1654-9)
Supplement: Supplementary file 1 — Table S1. Pearson correlation between samples. (DOCX 13 kb) [file 12870_2019_1654_MOESM1_ESM.docx]

**Table S1.** Pearson correlation between samples.

|  | Fe0_1 | Fe0_2 | Fe0Cd_1 | Fe0Cd_2 | Fe50_1 | Fe50_2 | Fe50Cd_1 | Fe50Cd_2 |
| --- | --- | --- | --- | --- | --- | --- | --- | --- |
| Fe0_1 | 1 | 0.949 | 0.884 | 0.902 | 0.911 | 0.936 | 0.939 | 0.938 |
| Fe0_2 | 0.949 | 1 | 0.897 | 0.903 | 0.927 | 0.921 | 0.942 | 0.926 |
| Fe0Cd_1 | 0.884 | 0.897 | 1 | 0.959 | 0.879 | 0.877 | 0.909 | 0.894 |
| Fe0Cd_2 | 0.902 | 0.903 | 0.959 | 1 | 0.888 | 0.895 | 0.912 | 0.903 |
| Fe50_1 | 0.911 | 0.927 | 0.879 | 0.888 | 1 | 0.95 | 0.932 | 0.923 |
| Fe50_2 | 0.936 | 0.921 | 0.877 | 0.895 | 0.95 | 1 | 0.935 | 0.942 |
| Fe50Cd_1 | 0.939 | 0.942 | 0.909 | 0.912 | 0.932 | 0.935 | 1 | 0.955 |
| Fe50Cd_2 | 0.938 | 0.926 | 0.894 | 0.903 | 0.923 | 0.942 | 0.955 | 1 |
