# Supplementary material for: Comparative transcriptome analysis reveals gene network regulating cadmium uptake and translocation in peanut roots under iron deficiency
Source: BMC Plant Biol. 2019 Jan 21;19:35. doi: 10.1186/s12870-019-1654-9 (PMC6341601; doi:10.1186/s12870-019-1654-9)
Supplement: Supplementary file 3 — Table S3. The primers used in RT-qPCR analysis. (DOCX 15 kb) [file 12870_2019_1654_MOESM3_ESM.docx]

| Genes | Directions | Sequence (5’–3’) |
| --- | --- | --- |
| *Ahactin* | Forward CTGAAAGATTCCGATGCCCTGA  Reverse AACCACCACTCAAGACAATGTTACCA | |
| *AhABCC3* | Forward TTTCTTCGTTCCTTCGTCTCG  Reverse TCCCACAAACAGCAACCCTC | |
| *AhCAX4* | Forward TCAAGCGATTCGGGTTCC  Reverse CGCCATTGCCCAAGGTTT | |
| *AhHMA5* | Forward GGAATGGCGATAGGTGCTG  Reverse ACCGAAATCTTGTAGAAGGGAAT | |
| *AhIRT1* | Forward GTTCTCTGCCTTATTCACGCTCAT  Reverse GCCAACACTAACAACAACACCCAT | |
| *AhOPT3* | Forward CAAGAACCACGCCGATGAC  Reverse GGTGTTGAGGAAGATGAGGAGC | |
| *AhNRAMP3* | Forward AGGTTGAAAAAATGGATGAGAGC  Reverse TCAGAACATCTAACGATTGCTCAG | |
| *AhNRAMP5* | Forward TTACTCCCAAACTCAGTGGTCAAG  Reverse GTGGAGGAAGAGGTTGTGCG | |
| *AhYSL3* | Forward TATTTGGAGAACCAGAGGCAGC  Reverse CGCCAACGATACTGGAATGC | |
| *AhZIP1* | Forward ATGAGATTGCGGCGGTGT  Reverse GCATAGTTCACATAGTCCTCTCCAG | |
| *AhZIP5* | Forward TTCCTTAGCGATTCCAATGACACT  Reverse CTTCTTCCACCTCCACGACCTT | |
| *AhADH3* | Forward GACGCTTGGCGAGATCAACA  Reverse AACCGGACAACCACCACATG | |
| *Ah60S* | Forward ACAGTTGGTCCTCACTTCAG  Reverse GCTCATTTATGTAAGCTTCCCT | |

Table S3 The primers used for qRT-PCR analysis in this study
